# Evoked Electromyographically Controlled Electrical Stimulation

**DOI:** 10.3389/fnins.2016.00335

**Published:** 2016-07-14

**Authors:** Mitsuhiro Hayashibe

**Affiliations:** Institut National de Recherche en Informatique et en Automatique (INRIA), University of MontpellierMontpellier, France

**Keywords:** electrical stimulation, evoked electromyography, personalized stimulation, muscle activation control, electrode effect cancelation

## Abstract

Time-variant muscle responses under electrical stimulation (ES) are often problematic for all the applications of neuroprosthetic muscle control. This situation limits the range of ES usage in relevant areas, mainly due to muscle fatigue and also to changes in stimulation electrode contact conditions, especially in transcutaneous ES. Surface electrodes are still the most widely used in noninvasive applications. Electrical field variations caused by changes in the stimulation contact condition markedly affect the resulting total muscle activation levels. Fatigue phenomena under functional electrical stimulation (FES) are also well known source of time-varying characteristics coming from muscle response under ES. Therefore, it is essential to monitor the actual muscle state and assess the expected muscle response by ES so as to improve the current ES system in favor of adaptive muscle-response-aware FES control. To deal with this issue, we have been studying a novel control technique using evoked electromyography (eEMG) signals to compensate for these muscle time-variances under ES for stable neuroprosthetic muscle control. In this perspective article, I overview the background of this topic and highlight important points to be aware of when using ES to induce the desired muscle activation regardless of the time-variance. I also demonstrate how to deal with the common critical problem of ES to move toward robust neuroprosthetic muscle control with the Evoked Electromyographically Controlled Electrical Stimulation paradigm.

## 1. Challenges in transcutaneous (surface) electrical stimulation -an introduction

Electrical stimulation of the nervous system is a technique which is frequently used in physical therapy as it offers clinical diagnosis on neuromuscular activation, physiological investigation, and functional control of paralyzed extremities (Merletti et al., 1992). In diagnostic applications, it is used to ascertain the integrity of neuromuscular junctions and reflex loops, as well as the excitability of motor neuron pools, nerves, and muscle fibers (Merletti et al., 1992; DeLuca and Erim, 1994). In therapeutic treatments, continuous usage of electrical stimulation can help to maintain muscle volume and enhance blood circulation following lesions of the nervous system. It can prevent muscle atrophy due to non-use, which can readily occur in spinal cord injured patients, and also in stroke patients during the immobilization period. In orthotic treatments, electrical stimulation can be used to provide functional control of paralyzed muscles, which is called functional electrical stimulation (FES).

FES and neuroprosthetic muscle control have been used to compensate motor functions or produce movements in patients with complete spinal cord injury (SCI; Kobetic et al., 1997), as well as in stroke patients with other sensory-motor deficiencies such as drop foot syndrome (Liberson et al., 1961). In the late 1990s, progress in microprocessor technology provided the means for computer-controlled FES systems (Donaldson et al., 1997; Kobetic et al., 1997; Guiraud et al., 2006). Electrical stimulation strategies are wide-ranging, from implanted stimulation (Triolo et al., 1996; Johnston et al., 2003; Guiraud et al., 2006), spinal stimulation (North, 2008) to transcutaneous surface stimulation (Mangold et al., 2005). In real-world applications, transcutaneous surface stimulation (TES) is the most frequently applied technique for muscle and nerve activation despite the significant efforts made for implantable technology developments (Keller and Kuhn, 2008), simply because TES is an easier solution in practice. Electrodes are placed on the skin at locations where the underlying tissue is intended to be activated. Electrical current is injected through a pair of bipolar electrodes and generates a potential gradient over the targeted area. This artificially generated gradient depolarizes excitable tissue beneath the electrodes that serve as cathodes, thus activating the underlying muscles.

TES has already been popularly utilized for muscular massage purpose. Portable electrical stimulators for home use are widely commercially available. Impulses are generated by a device and delivered through electrodes on the skin in direct proximity to the muscles to be stimulated. TES has also been receiving increasing attention in the last few years because of its potential to serve as a muscle strength training tool for healthy subjects and athletes and as a preventive tool for partially immobilized patients (Maffiuletti et al., 2011). Strength training promotes neural and muscular adaptations that are complementary to the well-known effects of voluntary resistance training. These types of TES devices for muscular massage and strength training are technically equivalent to TES systems which are applied by clinical specialists for therapeutic and functional purposes.

Following the wide availability of TES, many people now have experience on testing transcutaneous surface electrodes for stimulation in massage or training settings. If one has experienced to use surface stimulation electrodes, he would likely have noticed that the muscle status under TES is sometimes suddenly changed by the electrode contact condition to the skin. This is indeed a universal problem in all TES applications. A slight contact condition change can markedly affect the real muscle activation status, even if the same stimulation input is constantly applied. This is related to the correct electrode placement issue, which can cause reduced muscle contraction, especially in dynamic muscle contractions (Keller and Kuhn, 2008). There have been many FES studies of TES applications, it ranges from foot drop correction (O'Keeffe et al., 2003; Azevedo Coste et al., 2014) to upper limb muscle control (Chan et al., 2009). However, in these TES papers, variations in actual muscle activation due to the electrode condition is normally ignored in real applications, as it is hard to deal with the time-variant muscle response issue. As many FES researchers/users frequently encounter muscle activation changes due to electrode effects in TES, it is not negligible level of effect in terms of muscle activation changes. This intra-subject variability in muscle activation under TES is due to electrode dependency. There is also inter-subject variability coming from the muscle strength differences between different subjects. An able-bodied subject's muscle response could be more substantial than the weak muscle response in motor-impaired subjects. This factor also limits the controllability of muscles by ES, a problem that should be addressed via personalized modeling for FES. In this perspective article, I introduce a way to capture muscle activation changes through the concept of Evoked Electromyographically Controlled Electrical Stimulation, which was previously developed to compensate for muscle fatigue variations under FES. I address these issues below by outlining the research studies that have been carried out so far:

1. Personalized Electrical Stimulation through Evoked Electromyography (EMG).
2. Muscle fatigue prediction and compensation in FES.
3. Muscle activation predictive control and cancelation of the stimulation electrode effect.

## 2. Personalized electrical stimulation through evoked EMG

The challenge in implementing the present FES system arises with the problem of how to process the high nonlinearity and complexity of the neuro-muscular system (Durfee, 1993; Riener, 1999). Another challenge concerns time-varying muscle dynamics due to physiological and biochemical factors (such as fatigue, reflexes), as these need to be compensated in order to augment FES applications. Subject-specific modeling and time-variance compensation are essential to improve the performance of motor neuroprosthetics beyond the current limited use. A use of a mathematical model can improve the development of neuroprosthetics by optimizing their functionality for individual patients (Riener, 1999).

To achieve a reliable stimulation pattern and compensate muscle property changes during FES, in Ferrarin et al. (2001) the authors suggested using model-based approaches involving a feedforward controller to improve the control performance. In Jezernik et al. (2004), a sliding model closed-loop control method was proposed to control shank movement. In another work (Ajoudani and Erfanian, 2009), classic sliding model control and a neural network were combined to control FES to track the desired knee joint trajectory. A Feedback Error Learning controller for FES was developed by applying an antireset windup scheme (Watanabe and Fukushima, 2010). In a recent work (Freeman, 2014), iterative learning control was applied to control joint angles via stimulation of an arbitrary set of muscles with a Hammerstein-type muscle-activation recruitment relation.

Most closed-loop FES systems addressed in previous works were established between the electrical stimulus and joint angle since it is more convenient to measure and process the joint angle than joint torque or muscle force. However, in order to take the immediate effect of muscle activity induced by FES control into account, introducing biofeedback into closed-loops should be considered for further FES development (Bruns et al., 2013; Hayashibe et al., 2015), thus contributing to individualized modeling and control to manage different muscle responses from each different subject.

As muscle contraction is induced by artificial stimulation in FES, the drawback of closed-loop control of joint motion is that the resultant motion may not only derive from stimulation but also from external forces (such as environmental contact and gravity). The motion is the result of both external forces and the muscle contractions activated by the stimulation. The motion itself may not be directly related to the stimulation inputs. A stimulation pattern based only on motion signals is likely to be unsafe and unreliable in this case. In addition, it should be kept in mind that the muscle is a very slow actuator in terms of motion control. Error feedback by position information thus can not be instantly compensated, contrary to electromagnetic motor control in robotics. The muscle response to the same stimulation input is also not very consistent due to the physiological time-variant muscle response. Thus, considering the usage of biosignal feedback such as EMG from the muscle itself is a natural way for taking those potential physiological changes into account. By taking advantage of EMG signals, an EMG-triggered FES control system was presented through a pattern recognition (Dutta et al., 2008). This addresses mainly the FES start timing issue by using remained voluntary contractions rather than adapting the stimulation pattern itself. Then it does not handle time-variant muscle responses under FES.

Evoked EMG (eEMG) offers a way to study the myoelectric features of neuromuscular activation associated with electrical stimulation. Motor units activated by electrical stimulation have synchronous activity, with the so-called M-wave present in the EMG signal. When processing FES-evoked EMG signals, stimulation artifacts that appear at the onset of each stimulation impulse, and which are much larger than Mwaves, must be dealt with. In order to retrieve the signal of interest, i.e., M-waves, suitable techniques such as the blanking window method should be implemented to remove stimulation artifacts. The eEMG signal was found to be highly correlated with FES-induced muscular torque under various stimulation situations (Chesler and Durfee, 1997), and a similar phenomenon was also found in an implanted FES SCI subject (Hayashibe et al., 2011b). Moreover, M-waves extracted from the eEMG can be an effective detector for tracking potential muscle fatigue (Heasman et al., 2000). A pioneer work (Erfanian et al., 1998) proposed a predictive model of muscle force production under an isometric percutaneous continuous FES system. After comparing the performance of force prediction from stimulation and from EMG, the authors suggested using measured EMG signals instead of stimulation signals to predict muscle torque. This study was mainly carried out to predict joint torque as a kind of muscle force sensor in FES, and it has not yet been really used to achieve systematic FES closed-loop control based on evoked EMG. In our team, a new control strategy, i.e., EMG-Feedback Predictive Control (EFPC; Zhang et al., 2013), was proposed to adaptively control stimulation patterns to compensate time-varying muscle state changes. This facilitates the prediction of the muscle response and then the system can respond to time-variant muscle state changes toward muscle-response-aware FES control. It was further implemented combined with a wireless portable stimulator (Toussaint et al., 2010) to achieve real-time FES control (Li et al., 2015).

## 3. Muscle fatigue prediction and compensation in FES

Muscle fatigue has been defined as a failure to maintain the required or expected force from a repeatedly activated muscle (Edwards, 1981). In voluntary contraction, a variety of biological and motivational factors contribute to muscle fatigue (Gandevia et al., 1995), such as reduced motor drive by the CNS, failure of peripheral electrical transmission, and failure of the muscular contractile mechanism. The rate of muscle fatigue during FES is much greater than that which occurs during natural contractions (Binder-Macleod and Snyder-Mackler, 1993). This fast fatigue phenomenon is complex and is not yet fully understood. Currently, it is understood as follows: (1) the inverse size principle, according to which artificial stimulation recruits the motor neurons from the largest to the smallest (Hamada et al., 2004), and the larger the motor neuron, the more fatigable the muscle fiber; (2) motor units are activated in a synchronized manner with artificial stimulation, which is different from asynchronous activation during natural contraction. This situation requires a much higher stimulation frequency in synchronous stimulation to achieve quasi-tetanic contraction. The high stimulation frequency causes fatigue; and (3) the constant order of recruitment, with fast fatigable motor units activated first, then slow fatigue-resistant motor units. Another factor related to fast fatigue with FES is that the fatigue resistance of paralyzed muscles decreases after injury (Pelletier and Hicks, 2011). Systematic fatigue monitoring is especially important in paraplegic patients suffering from a lack of sensory feedback from their paralyzed muscles, because it can be used to adjust stimulation to prevent failure. Second, force prediction is essential if the muscle force has to be used as feedback in closed-loop stimulation.

Various fatigue models have been drawn up, based on physiological and mathematical interpretation or fitting from experimental measurements. A biomechanical model was developed to predict the shank motion induced by FES (Riener et al., 1996). A five-element musculotendon model was developed to predict the force generation capacity of activated muscles, and a fatigue recovery function based on the metabolic profile was introduced (Mizrahi et al., 1997). In Cai et al. (2010), a Wiener-Hammerstein model was proposed to predict FES-induced muscle force in unfatigued and fatigued muscle, and the model was verified by stimulating Soleus in SCI patients.

Some researchers have attempted to predict force/torque variations with fatigue based on eEMG. An exponential function was proposed to predict the force of FES-activated quadriceps muscles from eEMG Peak-to-Peak (PTP; Mizrahi et al., 1994). PTP was suggested as a fatigue index at a constant cycling speed in (Chen and Yu, 1997). A close correlation between the EMG mean absolute value (MAV) and knee torque was found under continuous stimulation in paraplegic subjects (Erfanian et al., 1996). In their following work (Erfanian et al., 1998), they proposed a predictive model of muscle force production under an isometric percutaneous continuous FES system. A metabolic model was presented to predict force decline and recovery from EMG signals under intermittent condition (Levin and Mizrahi, 1999). In Ziai and Menon (2011), re-training the model was suggested to regain the high estimation quality lost as a result of degraded estimation accuracy over time. Therefore, the online estimation method proposed in this study is preferable to characterize muscle contraction dynamics for real-time FES control. However, the fatigue properties vary with different fatigue levels and recovery processes, which complicates the identification of the fatigue model. A fatigue model cannot work when the desired stimulation pattern is unknown in advance.

**Figure 1:**
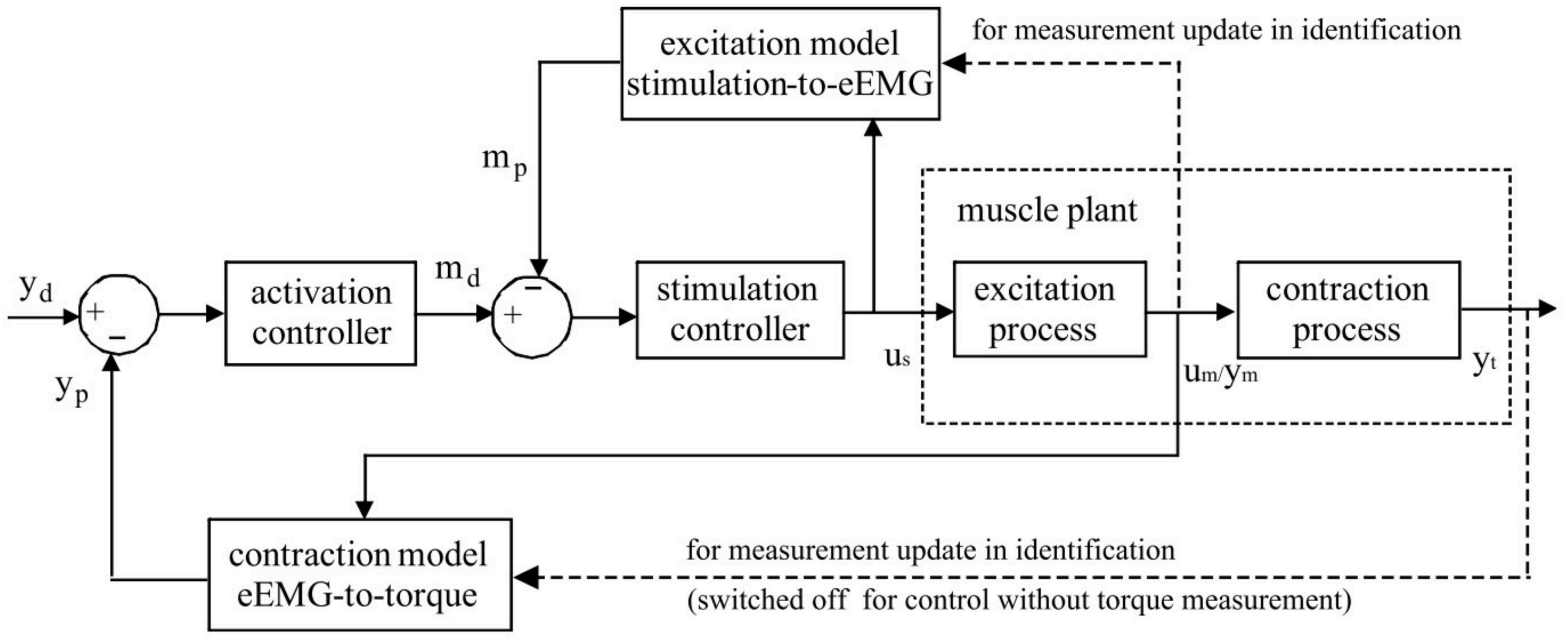
**Schematic representation of Evoked Electromyographically Controlled Electrical Stimulation**. Muscle activation is explicitly modeled as an intermediate variable between the stimulation input and the mechanical torque output. Muscle activation is obtained through evoked Electromyography under FES. Personalized modeling is performed for one block of muscular excitation process between stimulation and eEMG, and also for the other block of contraction process between eEMG and joint torque. Through the established models, the inversed solutions of stimulation input sequence could be systematically generated with model-predictive controllers to follow the desired mechanical output reference.

As in Figure 1, an online model estimation method is proposed for FES-induced torque prediction and muscle fatigue tracking through Kalman filter update (Zhang et al., 2011a,b). Muscle contraction dynamics modeled by a polynomial Hammerstein model (PHM) to represent joint torque from muscle activity based on evoked EMG, is updated systematically through Kalman filter with forgetting factor to correspond to the time-variant muscle contraction dynamics. This is further extended toward robust estimation with a nonlinear autoregressive with external input (NARX) model-based recurrent neural network (RNN) to predict torque with evoked EMG (Li et al., 2014b). The computational efficacy also makes it feasible for real-time implementation (Li et al., 2014a, 2016).

On the basis of the good predictive performance of the proposed estimation method, a new control strategy, i.e., EMG-Feedback Predictive Control, is proposed to explicitly control joint torque under FES (Hayashibe et al., 2011a; Zhang et al., 2013). Both muscle excitation and contraction dynamics are modeled by PHM. The eEMG signal is used for a dual-purpose: to correlate stimulation with the muscle electrical behavior in the muscle excitation process and to correlate the muscle electrical behavior with the muscle mechanical behavior in the muscle contraction process. Evoked EMG signal was used to feedback actual muscle states to track desired joint torque while considering the time-variant muscle dynamics. The EFPC control problem was resolved as a solution of two Nonlinear Predictive Control problems in series corresponding to activation and stimulation controller, respectively, as shown in Figure 1. The personalized models for excitation and contraction processes are developed for each subject for the model predictive controller to compute the inversed solutions. The activation controller solves the necessary muscle activation pattern to track the desired mechanical output reference. The stimulation controller solves the necessary stimulation sequence to achieve the necessary muscle activations. Once the torque deviates from the desired trajectory due to the effects of variations in muscle states such as fatigue, the controller adaptively generates the appropriate stimulation pattern in a systematic way to achieve the desired torque as long as it does not conflict with the stimulation constraints. This control framework provides satisfactory control accuracy and notable robustness in terms of joint torque control in FES (Zhang et al., 2013). This control strategy has a capacity to perform muscle fatigue compensation benefited from evoked EMG feedback.

## 4. Muscle activation predictive control and cancelation of the stimulation electrode effect

This section presents muscle activation closed-loop FES control through evoked EMG. It can be regarded as a simple version of EFPC regarding stimulation and muscle activation only.

This muscle activation control can present an advantage of Evoked Electromyographically Controlled Electrical Stimulation. In conventional FES, we normally specify the stimulation pattern and there is no way to check if the muscle is really responding as needed. As the electromagnetic motor is normally observed through the encoder, it is advantageous to monitor the reaction of muscles to electrical stimulation. Since evoked EMG can always be observable through the acquisition system, eEMG could be used for updating the stimulus-to-eEMG model. This could improve the modeling precision of the plant and guarantee the accuracy of the predictive model controller.

A real-time implementation of the predictive model controller for online control of muscle activation is as follows (Li et al., 2015):

1. The reference muscle activation trajectory is prepared before beginning estimation and control;
2. Trapezoidal shape pulse width stimulation at different amplitude levels is tested while recording the eEMG to personalize the model regarding the relationship between the pulse width and MAV of eEMG via Kalman filter;
3. After the identification phase ends, the FES system goes into control mode. The stimulator is driven by a predictive controller to modulate the pulse width to track the desired muscle activation trajectory while the stimulation-to-eEMG model is being updated to correspond to the time-variant properties.

**Figure 2:**
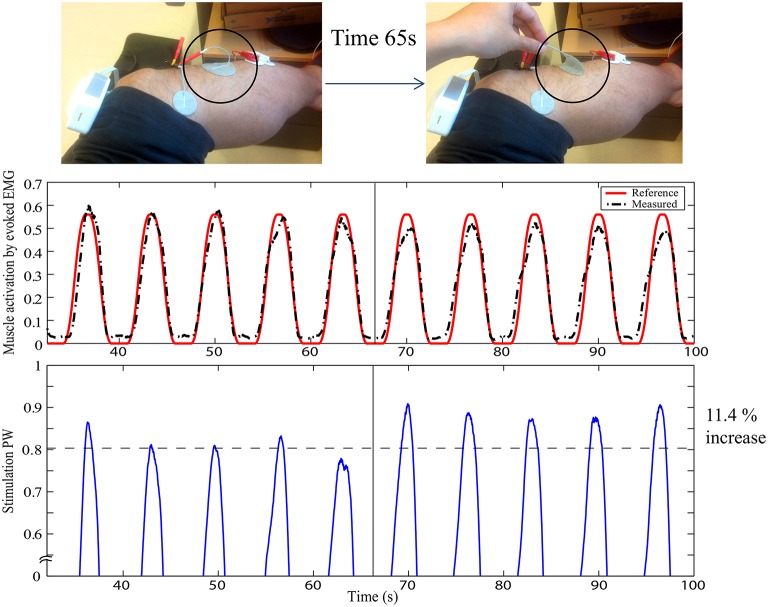
**Stimulation electrode effect cancelation in FES: The upper figure represents the experimental setup with typical foot drop correction montage for the stimulation electrode to stimulate the peroneal nerve for the dorsi flexion of the ankle**. EMG is measured for the tibialis anterior muscle. At time 65 s, one electrode is detached, as indicated by the circle. The first plot shows the muscle activation obtained by evoked EMG. The second plot shows the stimulation pulse width. Y-axes are in normalized scale. Note that the stimulation is systematically modified to compensate for the effect of the electrode detachment. Muscle activation could thus still be maintained to minimize the effect of the stimulation field variation.

In this way, muscle activation can be fully specified in the framework of Evoked Electromyographically Controlled Electrical Stimulation, instead of specifying the stimulation parameters. This can be useful to compensate for time-variant processes in FES. Figure 2 shows an example of cancelation of the stimulation electrode effect. At the time 65 s, part of the electrode is suddenly detached without informing it to the system, as indicated by the circle. Muscle activation can be easily influenced by this situation. However, as the change is observed with evoked EMG signal from the targeted muscle (tibialis anterior), the controller systematically modifies the stimulation input to be increased so as to track the desired reference pattern of muscle activation. It is pulse width control then it may not fully cancel the effect but it could minimize the effect of sudden muscle activation changes by the detachment or the contact condition changes of the electrode. The stimulation electrodes are with typical montage for foot drop correction by FES.

## 5. Conclusion

Transcutaneous electrical stimulation (TES) is the technique most frequently applied for muscle and nerve activation. It is known that it has high sensitivity to the electrode contact condition for electrical stimulation. To compensate the intersubject (muscle strength) and intrasubject variability (stimulation electrode contact change, muscle fatigue), it is essential to monitor the real muscle responses under FES. Evoked Electromyographically Controlled Electrical Stimulation could be one solution to deal with this important issue. This framework contributes to augmenting the FES system in several aspects. First, an appropriate personalized muscle response model under FES (stim-emg, emg-torque) could be quickly established. Prediction of joint torque affected by muscle fatigue could be performed based on evoked EMG. This allows us to systematically compute and control electrical stimulation so as to achieve the desired muscle activation even under time-variant disturbances such as muscle fatigue and contact changes of stimulation electrode. An example of stimulation electrode effect cancelation was demonstrated to show the promising performance of Evoked Electromyographically Controlled Electrical Stimulation, which enables us to induce stable muscle activation in TES.

## Author contributions

MH wrote the perspective article along with previous research activities he has performed as a PI of this work.

### Conflict of interest statement

The author declares that the research was conducted in the absence of any commercial or financial relationships that could be construed as a potential conflict of interest.
